# BoxStacker: Deep Reinforcement Learning for 3D Bin Packing Problem in Virtual Environment of Logistics Systems

**DOI:** 10.3390/s23156928

**Published:** 2023-08-03

**Authors:** Shokhikha Amalana Murdivien, Jumyung Um

**Affiliations:** Department of Industrial and Management System Engineering, Kyung Hee University, 1732 Deogyeong-daero, Yongin-si 17104, Republic of Korea; amalanadivien@khu.ac.kr

**Keywords:** Deep Reinforcement Learning, bin packing problems, robot scheduling, warehouse systems, logistics, artificial intelligence

## Abstract

Manufacturing systems need to be resilient and self-organizing to adapt to unexpected disruptions, such as product changes or rapid order, in supply chain changes while increasing the automation level of robotized logistics processes to cope with the lack of human experts. Deep Reinforcement Learning is a potential solution to solve more complex problems by introducing artificial neural networks in Reinforcement Learning. In this paper, a game engine was used for Deep Reinforcement Learning training, which allows visualization of view learning and result processes more intuitively than other tools, as well as a physical engine for a more realistic problem-solving environment. The present research demonstrates that a Deep Reinforcement Learning model can effectively address the real-time sequential 3D bin packing problem by utilizing a game engine to visualize the environment. The results indicate that this approach holds promise for tackling complex logistical challenges in dynamic settings.

## 1. Introduction

In modern manufacturing systems, establishing resilience is crucial to adapt to unforeseen disruptions, such as sudden product alterations or rapid fluctuations in supply chain dynamics. Concurrently, as these systems aim to augment automation through robotized logistics processes, this becomes particularly crucial in compensating for potential shortages in human expertise [1]. The COVID-19 pandemic has resulted in a surge of activity in the delivery and logistics industry, as social distancing measures have necessitated the increased use of robotized logistics systems. Due to unexpected disruptions, such as product changes or rapid orders, re-planning of the robotized handling system is required frequently for complex loading and unloading tasks, which are intensive manual labor. Overall, the tasks remain reliant on manual labor, which is the least automated part of the entire logistics process. Previous loading problem solutions only focus on how to load a container, whereas bin packing problems (BPP) are handled separately [2]. However, since the picking position of the box impacts the area in which the box can be placed, those procedures should be planned concurrently to optimize space [3]. Since the courier logistics environment is a variable and dynamic environment, there is a limit to human design and automation of robot work.

In recent years, significant breakthroughs have been achieved through Deep Reinforcement Learning (DRL) to address these challenges. DRL is actively studied in artificial intelligence academia, with a particular focus on optimal robot control. By introducing artificial neural networks to Reinforcement Learning, DRL enables learners to strengthen their behavior through function approximation methods, even for initially unsolvable problems. Based on feedback during the training process, the agent decides actions from a set of available options to optimize the collected reward. DRL has the potential to learn algorithms for complex behavior using rewards, making it applicable to develop robotic models for loading and unloading tasks in courier logistics environments.

The high dimensionality of 3D box stacking poses significant challenges that legacy heuristic algorithms cannot effectively handle. While Reinforcement Learning shows promise in solving such problems, the curse of dimensionality limits its practical application to larger problems. This limitation hampers the real-world implementation of Reinforcement Learning for addressing practical challenges. DRL algorithms can efficiently process large inputs, enabling rapid and effective learning in systems. However, their performance often deviates from reality. To bridge this gap, this study aims to develop an artificial intelligence agent using game engines to construct a virtual delivery environment and simulate delivery loading. Using a game engine to perceive the environment, a Deep Reinforcement Learning model successfully addresses the real-time sequential 3D bin packing problem.

This paper tries to provide approaches to practical challenges and answer the following research questions:

1. How is the courier logistics problem of loading and unloading boxes solved? The evaluation process is carried out by applying Reinforcement Learning to develop the bin packing algorithm.
2. How can solutions that can perform similarly to reality be provided? This will be solved by building the experiment environment with a 3D game engine.
3. To what degree are the proposed solutions optimal? This will be analyzed by evaluating the feasible solutions made by DRL and finding the best positions for packed boxes.

This paper is organized as follows: Section 2 provides reviews of the relevant literature and an overview of DRL and 3D game engine technology. In Section 3, the environments and learning functions of Reinforcement Learning for stacking boxes are proposed. The obtained results are presented and analyzed in Section 4, followed by discussions in Section 5. In the final section, the conclusions of the research are summarized.

## 2. Literature Survey

This section will provide an overview of earlier research that utilized Deep Reinforcement Learning and, in particular, game engines, to solve the bin packing problem.

### 2.1. Bin Packing Problem

The bin packing problem (BPP) is a well-known and essential combinatorial optimization problem in logistics and manufacturing and a huge number of methods exist in the literature for packing problems [4,5]. The most crucial and challenging is the 3D bin packing problem, in which a variety of cuboid-shaped objects of various sizes must be packed orthogonally into bins [6]. The 3D BPP is a real-world-driven combinatorial optimization problem with significant economic, environmental, and safety implications [7]. The geometric 3D BPP is a variation of the knapsack problem and is a well-known NP-hard problem in computer science literature [8]. The purpose is to create a packing method for boxes of various shapes and sizes that maximizes the space within the bin. Thus, an efficient bin packing algorithm reduces calculation time, overall packing cost, and resource consumption.

Many researchers have suggested various approximations or heuristic algorithms [2,5] due to the difficulties in achieving optimum bin packing problem solutions, including integer linear programming [9], space minimizing heuristics [10], genetic algorithms [11,12], quantum algorithms [13,14] and machine-learning-based smart heuristic selection [15]. Solving bin packing in a 3D environment is not an easy task. It has multi goals, different box sizes, and has to consider orientation and stability [6]. Load stability and balance factors are generally not considered explicitly in the bin packing literature [16].

### 2.2. Deep Reinforcement Learning in 3D BPP

Recently, the bin packing problem has been solved through Deep Reinforcement Learning (DRL) [17,18,19,20,21]. Reinforcement Learning (RL) is a field of machine learning that entails a set of techniques for determining the optimum agent strategy and maximizing the reward of the agent [22]. DRL uses a deep neural network (DNN) to extend Reinforcement Learning without having to explicitly define the state space. Therefore, it combines both RL and Deep Learning (DL). Artificial intelligence, particularly Deep Reinforcement Learning, has gained a lot of attention in recent years and has produced outstanding breakthroughs in a variety of sectors. Furthermore, the DRL technique has shown great promise in solving combinatorial optimization issues [23,24]. Hu et al. applied the Traveling Salesman Problem and Pointer Network to solve the 3D bin packing problem [6]. Kundu et al. used 2D vision-based Reinforcement Learning to pick a bin [17]. Le et al. not only applied bin packing but also considered conveyor belt movement and placing in the RL [18]. Bo et al. transformed the online 3D palletization problem into partially observable Markov decision processes (POMDPs) and introduced the DRL technique to estimate states using observation trajectories [25].

### 2.3. Reinforcement Learning in 3D Game Engine

DRL algorithms need a huge number of experiences to be able to learn complicated tasks. In order to teach the agents, speed up their learning, and function properly in the real world, a realistic simulation environment must be performed; thus, it requires advanced programming knowledge. Therefore, a physics and game engine capable of supporting multi-agent systems can be the solution to address this issue. A physics engine simulates physical interactions (e.g., PyBullet [26], MuJoCo [27]), while a game engine is a versatile software framework for game development (e.g., Doom [28], Unreal [29]), including graphics, physics, AI, and networking capabilities [30]. In this paper, the game engine was used to visually demonstrate the 3D environment and the physical engine was used to conduct DRL in a more realistic environment. Unity3D is one of the most well-known virtual reality technologies and is a cross-platform game production program [31]. Unity has the capacity to generate diverse multi-agent environments [32]. While there are several works that address 3D online bin packing problems using Deep Reinforcement Learning (DRL) [33,34,35], as of now, there is no work dealing with the autonomous management of loading and unloading boxes using Deep Reinforcement Learning and game engines to perform the simulation. This presents an opportunity for further research in this area, as the incorporation of a game engine may provide additional flexibility and capabilities for training and evaluating DRL algorithms for the 3D BPP.

### 2.4. Summary and Opportunities

The following conclusions were obtained from the literature review:

1. Three-dimensional box stacking has high-dimensional problems which cannot be solved by legacy heuristic algorithms. This method had the disadvantage that it may become impractical to use when the size of the problem becomes too large due to the “curse of dimensionality”. This can make it challenging to apply these methods to real-world problems.
2. Even though DRL can provide available solutions, its performance is often far away from reality in many cases.
3. Using open space made by a physical game engine to give a degree of freedom to all arbitrarily shaped boxes is the challenge of the previous literature.
4. Simple modeling of 3D bin packing is better for adapting to new configurations of warehouse problems.
5. A more realistic environment of bin packing is helpful to realize realistic situations without statistical simulation.

The aforementioned observations indicate an opportunity to motivate Reinforcement Learning for open space-based 3D bin packing. In addition to a clear need for an alternative technological solution in this area, the environment development and reward function using game-engine-based system architecture will provide benefits in the development of innovative algorithms for various conditions.

## 3. Methodology

### 3.1. System Specifications

**Figure 1:**
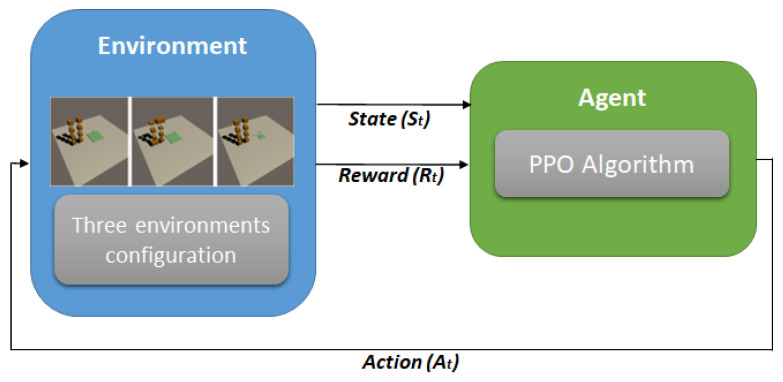
General structure of Deep Reinforcement Learning concept in proposed work.

The procedure to build a Reinforcement Learning environment in game engines is as follows: defining agents (observation, action), rewards, and selecting algorithms. Figure 1 depicts the structure of the DRL concept in the proposed work. It consists of three dynamic environment settings and an agent. The environments are the layout of warehouses in a simplified manner, and the agent is the box that should learn how to load the box into the designated plate, which is the simulation of the space inside the container.

In order to design a Reinforcement Learning environment and an agent, it is recommended to go through the following process:

1. Define goals or specific tasks to be learned.

**Figure 2:**
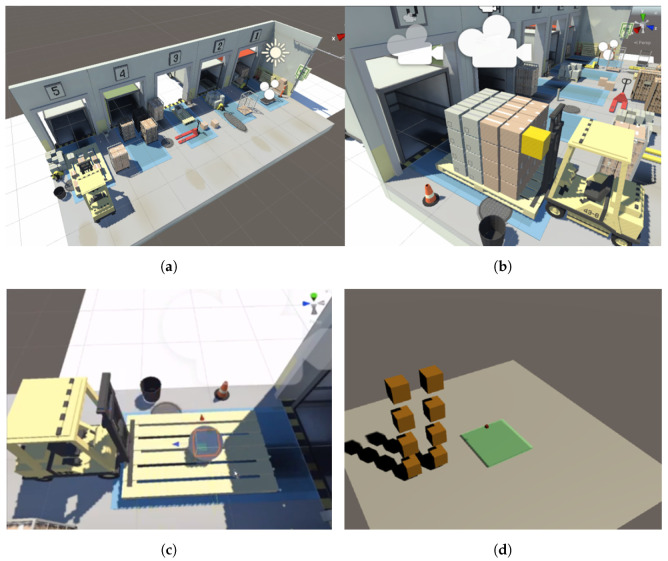
Proposed work virtual environments in all cases. (**a**) All objects of the warehouse, (**b**) all objects of each slot, (**c**) possible movement of each box, and (**d**) the simplified loading plate.

The goal of this study is to train an agent to stack boxes on a designated plate area, with the goal of ensuring that the boxes are centered on the center of the plate. The training process is conducted in a simulated warehouse environment that is designed to be visually similar to a real-world setting. The warehouse contains five slots, but for the purpose of the training, only one slot is used, as all the slots are identical. This allows for more efficient training by eliminating the need to consider variations between slots. To facilitate the observation of the learning process, the training of the boxes is initially conducted in a simplified environment that consists of only eight boxes. This allows for a clearer understanding of the learning dynamics without the added complexity of a larger number of boxes. The proposed work environment settings are shown in Figure 2.

2. Define agents.

The agent is the one that acts in Reinforcement Learning. The agent may be an actual object or an abstract object inside the Reinforcement Learning environment. In the context of this study, the agent is represented by a small box placed in the center of the plate. Through the process of Reinforcement Learning, the agent will learn to determine the optimal position for placing the real boxes on the plate one by one. Since the agent has to act directly and achieve its goals, an agent script to an actual object was assigned.

3. Define agents’ behavior and observation.

**Figure 3:**
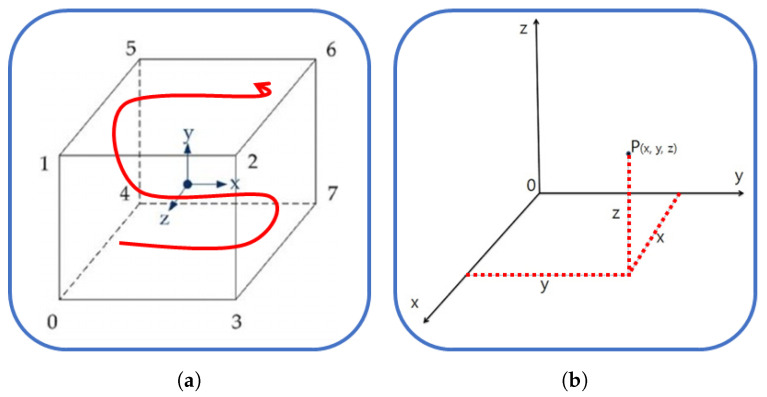
Problem complexity of (**a**) movement to X, Y, and Z coordinate axes, and (**b**) movement to X and Y coordinate axes.

In the learning environment, the agent must define what information and behavior to observe. The Reinforcement Learning model uses observation information as an input of the model and outputs the value of the state or behavior. In this study, the agent generates a box (object which needed to load). Generating a box is the action of the agent. Agent observes the generated box’s positions and how many boxes are stacked. The agent receives rewards if all the layers are stacked. In the initial stages of this research, the agent was programmed to move in three dimensions (x, y, and z axes) during the learning process, as shown in Figure 3a. However, the complexity of the problem was found to be excessively high (approximately O(6^1000000^)), due to the infinite trajectory space. As a result, the agent was subsequently modified to generate movement only on the x and y axes (Figure 3b), reducing the complexity of the problem (O(4^1000000^)).

4. Define the reward.

**Figure 4:**
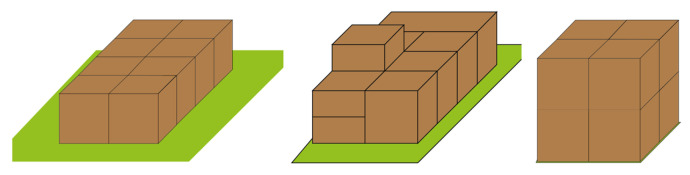
Optimal solution for each scenario, the figure on the left is boxes of the same size, the middle is boxes of varying size, and the right figure is small plane environment.

Next, and the most important, the reward for Reinforcement Learning should be defined. No matter how well the environment and agent are designed, the agent cannot learn what the target is unless a reward is given. The reward should be provided when the agent acts and satisfies certain conditions. At this time, the amount of the reward and the timing of reward are important. Determining the informativeness of a reward function is an essential issue [36,37]. The agent will get a reward after being able to stack the boxes, and after all the boxes are stacked, an evaluation will be made of which solution is close to the optimal solution, as illustrated in Figure 4. The reward function used in this study can be seen in Algorithm 1.
**Algorithm 1:** Box Stacker Agent Learning
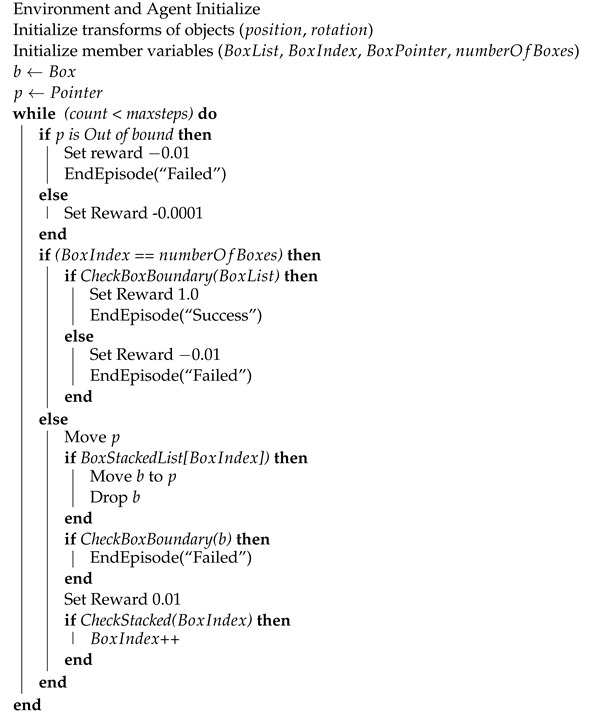

5. Define the conditions of the start and end of the episode.

The initial state and termination conditions must be set at the beginning of the episode. The end of the episode is divided into success and failure in achieving the goal, and rewards for success and failure must also be allocated. At the end of the episode, the episode begins again, and at this time, learning begins again to its initial state. If the initial state (agent location, environment information) is set randomly, learning takes longer, but more general performance can be expected. In this study, the environment consists of a plane for stacking boxes, a thin collider surrounding the plane, and an agent. The agent’s goal is to create a target point within the specified range of the plane and then place a box at that point. If the box collides with a virtual wall or goes out of bounds during the process of placing it, the agent receives punishment, and the episode is terminated. Therefore, the stacking attempts will restart from the first box again. However, if all the boxes are placed without collision, the agent is rewarded for successfully stacking one floor. The agent inputs the information of the changed environment into the observation and then creates a new coordinate to place another box at the target point. This process is repeated until the agent either succeeds in stacking all the boxes or receives punishment for an unsuccessful attempt.

6. Reinforcement learning model (algorithm) selection.

The Reinforcement Learning algorithm is divided into a value-based algorithm and a policy-based algorithm. In this case, if there is a probabilistic element in the environment, it is recommended to use a policy gradient-based algorithm. In general, policy gradient-based algorithms perform better in environments such as robot control. A detailed explanation about algorithms used in this study will be explained in the next subsection.

### 3.2. Algorithm

The Proximal Policy Optimization (PPO) Algorithm by Schulman et al. is a prominent policy gradient algorithm for solving the optimization problem [38], and has proven to learn policies more efficiently than Trust Region Policy Optimization (TRPO) [39]. From past studies, this algorithm achieved better overall performance while also ease of implementation and hyperparameter tuning to achieve even better results. PPO performs each policy update over numerous epochs of stochastic gradient ascent. In the case of ML-Agents, PPO is recommended since it provides more stable results in the environment and has better generalization ability [40]. Therefore, PPO algorithms are chosen to implement the model in this study.

After initializing the policy parameters, θ, it collects a batch of transitions (*s_t_*, *a_t_*, *r_t_*, *s_t_*^${}^{\prime }$^) from the environment using the current policy. Then, it estimates the expected reward gradient in relation to the parameters of the policy as follows:

(1) $$\nabla_{\theta }J\left(\theta \right)=\frac{1}{N}\sum_{t_{\theta }^{\nabla }log}\pi_{\theta }\left(a_{t}|s_{t}\right)*A_{t}\left(s_{t},a_{t}\right)$$

where *N* is the number of transitions in the batch, $\pi_{\theta }$$\left(a_{t}|s_{t}\right)$ is the probability of taking action $a_{t}$ in state $s_{t}$ according to the current policy, and $A_{t}$$\left(s_{t},a_{t}\right)$ is the advantage function for the *t*-th transition. Then, compute the PPO objective function:

(2) $$L_{CLIP}\left(\theta \right)=min\left(r_{t}\left(\theta \right)*\nabla_{\theta }J\left(\theta \right),clip_{param}*\nabla_{\theta }J\left(\theta \right)\right)$$

where $r_{t}\left(\theta \right)$ is the ratio of the new and old policy probabilities and $clip_{param}$ is a hyperparameter that limits the size of the policy updates. Lastly, perform a gradient ascent step on the PPO objective function:

(3) $$\theta =\theta +\eta *L_{CLIP}\left(\theta \right)$$

where η is the learning rate. This update step is repeated until the policy has converged.

### 3.3. Workflow of Deep Reinforcement Learning

Algorithm 1 outlines the process of training an agent to stack boxes in this study. The training process involves moving the agent, represented by a box pointer, to stack the boxes. If the current box is out of bounds or successfully stacked, the agent receives a reward, and the current box is moved. The training process is terminated when the index for the current box reaches the total number of boxes, at which point the episode is marked as a success or failure depending on whether all the boxes were stacked. The agent receives a positive or negative reward in each case. The training process is terminated when the maximum number of steps is reached.

**Figure 5:**
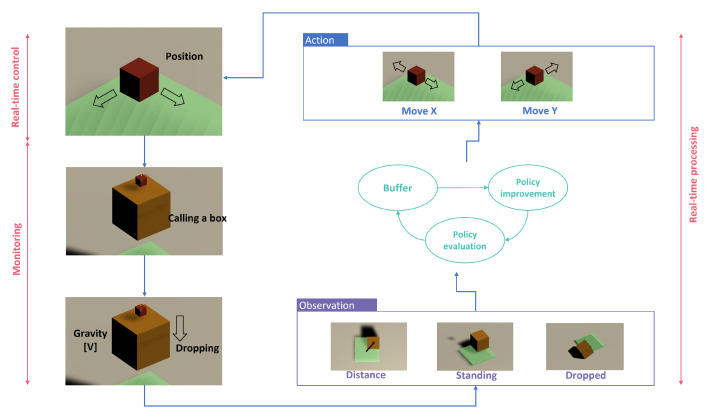
Training process of Reinforcement Learning.

Basic objects of Reinforcement Learning are a plate to support the dropping boxes, a pointer to call each box during flying over the plate, and boxes waiting for loading on the plate. The agent of the pointer reports observations and rewards after conducting actions generated by the PPO algorithm, as shown in Figure 5. First, the pointer moves along the X and Y axis. Next, each box is transferred to the position of the point and dropped when gravity is applied to the box. The agent checks if the box stands on the plate or drops out. The reward is a plus value when standing while a minus value when dropping out.

The reward of each agent action is the distance of each box from the center of the plate step by step, as well as the final status of the dropped box. In addition, the PPO algorithm will be used for training, with the following hyperparameter settings, as shown in Table 1.

## 4. Experiment and Results

### 4.1. Experimental Planning

**Figure 6:**
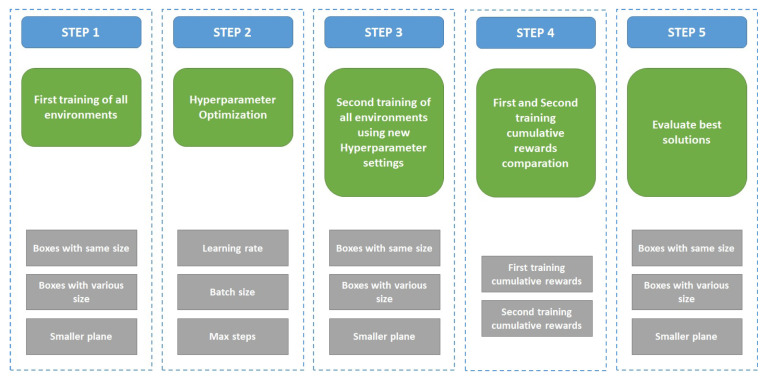
Overall architecture of the experiment.

DRL is applied to find feasible solutions and add the evaluation phase to find the best success results among the feasible solutions. Especially in this study, DRL with a learning process that generates random positions for the box and results in a high degree of freedom is being proposed. Hence, the combined results of the learning process are much bigger. Therefore, the next evaluation step is needed. The evaluation phase consists of visualizing the successful solutions during the training process, calculating the summation of the gaps between the center position of the bottom plane and each box, and finding the best solution among the successful solutions. As well as the comparison of the summation of gaps, the performance of Reinforcement Learning was compared with different hyperparameters such as batch size, max steps, and learning rate. The overall architecture is presented in Figure 6. There are five steps, starting with the first training of all environments that are boxes with the same sizes, boxes with various sizes, and the smaller plane environment. The second step is hyperparameter optimization with the main focus on learning rate, batch size, and max steps parameters. The next step is the second training of all environments using new hyperparameter settings followed by cumulative rewards cooperation of first training and second training. Lastly, solutions are evaluated to find the best one.

In this experiment, Unity version 2018.4.31f1 and ML-Agents 0.16.0 has been used. Compared to other game engines, when it comes to an open simulator such as Mujoco, Gazebo, and Bullet, the model input is more complicated and poses challenges for ease of use and implementation. While the Unreal Engine excels as a powerful game engine, it currently lacks a native RL toolkit and users have encountered difficulties in establishing connections with external modules. Unity offers the advantage of built-in ML-Agents for RL and seamless integration with external tools, making it a more user-friendly option for developers seeking to incorporate Reinforcement Learning into their projects. The training was conducted on a computer with an Intel(R) Core(TM) i5-8500 CPU running at 3.00 GHz, 8 GB of RAM, and an NVIDIA GeForce RTX 3060 GPU.

### 4.2. Experiment Scenarios

**Figure 7:**
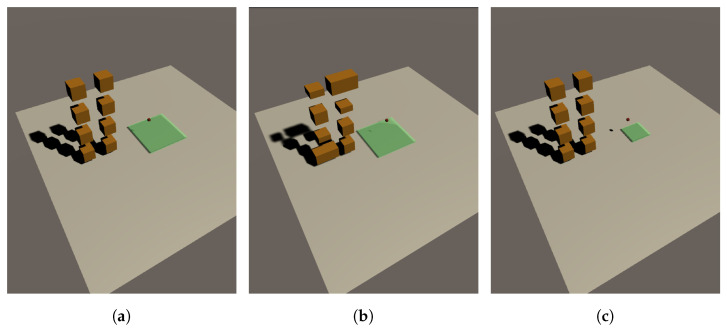
Three environment scenario configurations, (**a**) box of same size, (**b**) box of varying size, and (**c**) small plane.

In this study, three experimental conditions depicted in Figure 7 were conducted in order to examine the effect of different factors on the outcome. The first condition involved the use of boxes of the same size, while the second condition involved the use of boxes of varying sizes. The third condition involved reducing the size of the plane. For each condition, multiple training was with different hyperparameter settings in order to analyze the results and identify trends.

### 4.3. Hyperparameter Tuning

In general, max steps, learning rate, and batch size are important hyperparameters that can have a significant impact on the performance of the model. The appropriate values for these hyperparameters will depend on the characteristics of the dataset and the complexity of the model. Experimentation and tuning may be necessary to determine the optimal batch size for a particular model and dataset.

Max steps in ML-Agents is a hyperparameter that defines the maximum number of steps that the environment will be allowed to run throughout each training episode. The step size at which the model updates its weights during training is controlled by another hyperparameter called the learning rate. It is a parameter of the optimizer, which is the algorithm used to adjust the model’s weights based on the calculated gradients. The learning rate determines the size of the update that the optimizer will make to the model’s weights at each step of the training process. Meanwhile, batch size determines the number of training examples that will be used in each iteration of the training process.

**Figure 8:**
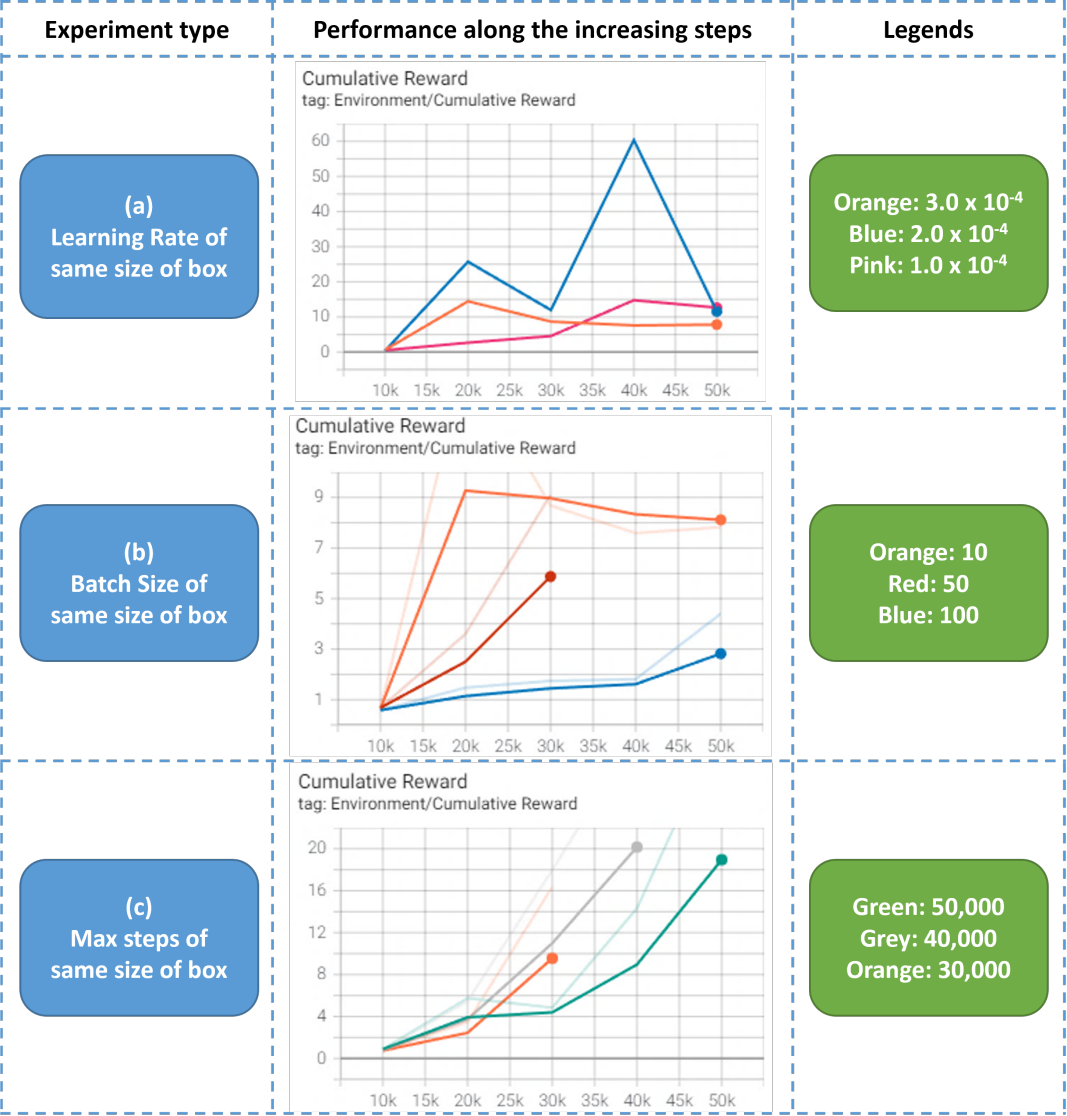
Training results of various experiment types.

In this study, experiments were carried out for the max steps, learning rate, and batch size parameters to get the optimal parameters to train the model. An experiment was conducted to evaluate the effects of various learning rates (1.0 × 10^−4^, 2.0 × 10^−4^ and 3.0 × 10^−4^), batch sizes (10, 50, and 100), and maximum number of steps (30,000, 40,000, and 50,000) on the performance of a Deep Reinforcement Learning algorithm. The results of this experiment can be used to identify the optimal set of hyperparameters for this problem. The comparisons of experiments are shown in Figure 8.

The results of the hyperparameter tuning suggest that the optimal combination for the learning rate, batch size, and maximum number of steps for the Deep Reinforcement Learning algorithm were 2.0 × 10^−4^, 10, and 40,000, respectively, based on the highest cumulative rewards achieved. These findings may be useful for guiding the selection of hyperparameters in future applications of the algorithm to the 3D bin packing problem.

### 4.4. Cummulative Rewards Comparation

The agent was retrained using the optimized hyperparameter setting (Table 2), and the resulting cumulative rewards were compared to those obtained during the first training with the old parameter setting. The results indicate that the new parameter setting resulted in higher cumulative rewards (Table 3). Boxes with the same size obtained 7.823 cumulative rewards, 5.866 for boxes with different sizes, and 56.03 for smaller plane using the old hyperparameter settings. Meanwhile, by using the new hyperparameter settings, boxes with the same size acquired 12.66 cumulative rewards, boxes with different sizes received 6.101, and the smaller plane earned 238.1. This suggests that the optimization of the hyperparameters can significantly improve the performance of the Deep Reinforcement Learning algorithm for the 3D BPP.

### 4.5. Evaluation

**Figure 9:**
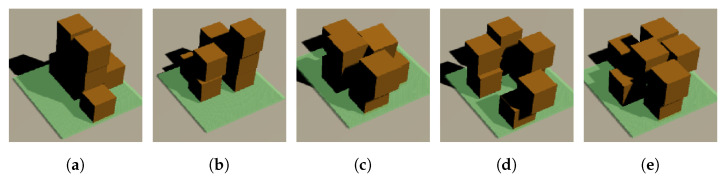
Success results visualization; (**a**) Solution 1; (**b**) Solution 2; (**c**) Solution 3; (**d**) Solution 4; (**e**) Solution 5.

The final stage of the training process involves the evaluation of the results. If all the boxes are successfully stacked on the plate, a success list is generated. This list includes the position of each box on the x, y, and z axes. To determine the best or near-optimal solution, the total gaps between the center point of each box and the center point of the plate are calculated. The solution with the smallest gaps is considered to be the best solution from the training process. However, due to the large number of success lists generated from the training process, as an example, only five success lists for boxes of the same size are shown (Table 4). The visualization of the success list results is also presented in Figure 9.

As demonstrated in the table, Solution 3 exhibits the lowest total gap among the five solutions presented with the value of 5.7502. Therefore, it can be concluded that Solution 3 is the optimal solution among the ones shown in the table.

## 5. Discussion

The algorithm for loading packages can be implemented in several ways. Theoretically, this problem can be infinitely improved. However, there are limitations to trying various methods because it consumes a lot of training time and a lot of computing resources in reality. In this study, training using various hyperparameter configurations was implemented in order to identify the optimal settings for the proposed model. As expected, the training process of boxes with different sizes was the most challenging condition, as evidenced by the lower cumulative rewards observed in comparison to the other conditions.

The reward function of the proposed model focuses on the gap from the center position demonstrating a valuable aspect in promoting balance and stability during box stacking on a pallet. By encouraging the agent to prioritize the center position, the reward function fosters successful stacking attempts and reduces the likelihood of the boxes toppling over. To optimize the reward function and facilitate comprehensive learning, it is essential to incorporate other factors in the reward function, such as the height of stacking, collision avoidance, and efficient space utilization, that contribute to stable and efficient stacking, ultimately guiding the agent toward mastering the box stacking task successfully. A balanced combination of these elements, including maintaining the center position, will empower the agent to achieve the best solution by creating stable, efficient, and well-distributed stacks on the pallet.

The use of a game engine allows for the development of a user interface that makes it simple to change the size of the box and the plate even for users who are not programmers. Additionally, this approach is applicable to dynamic environments since it also has the ability to generate various shapes other than only cuboids.

This study presents a novel approach utilizing the Unity game engine to address the 3D BPP. The method achieves smoother and more efficient packing roll-outs due to the advanced features of the engine. Furthermore, improved stability in packing results is observed, leveraging the engine’s robust physics simulation and collision detection capabilities. Unlike conventional heuristics and RL approaches without game engines, the proposed method accommodates arbitrary shaped objects, enhancing the flexibility of packing configurations. Additionally, the visualization capabilities offer clear and intuitive representations of the packing process and outcomes, facilitating comprehensive analysis and evaluation. Moreover, the approach enables easy modification and adjustment of packing environments and parameters, streamlining experimentation and optimization. Overall, the study demonstrates the superior performance of the proposed method in roll-out efficiency, stability, handling arbitrary shapes, visualization, and ease of modification compared to traditional techniques (Table 5).

## 6. Conclusions

This study demonstrated the use of Deep Reinforcement Learning to automate the loading process in a simulated environment. The combination of a learning rate of 2 × 10^−4^, a batch size of 10, and a maximum number of steps of 40,000 resulted in the best performance for the proposed model, suggesting that the optimization of the hyperparameters can significantly improve the performance of the DRL algorithm for the 3D BPP. However, limitations were identified in the reward function of the proposed work, which was insufficient for guiding the learning process to reach the optimal solution. Further improvements in the reward function are needed in order to fully optimize the proposed model. This approach has the potential to be a valuable tool for improving the efficiency of the loading and unloading process in the logistics industry. The current study shows that by visualizing the environment using a gaming engine, a DRL model may successfully handle the real-time sequential 3D bin packing problem. The game engine’s ability to visualize the packing process and outcomes allow for clear and intuitive representations, making comprehensive analysis and evaluation easier. The proposed method achieves smoother and more efficient packing roll-outs due to the advanced features of the engine. Moreover, improved stability in packing results is observed, leveraging the engine’s robust physics simulation and collision detection capabilities. The usage of a game engine in the training process, on the other hand, enables the use of various forms, including shapes other than cuboid boxes, such as cylinders. Additionally, the game engine facilitates the implementation of the digital twin system and enhances its capabilities through the integration of virtual reality technology. The utilization of a game engine thus provides a more advanced and flexible approach to training for the 3D BPP. The findings show that despite its limitations, the use of a game engine and its machine learning agents (ML-Agents) toolkit allows for the development of a user-friendly interface and has the potential to address complicated logistical difficulties in dynamic environments.

## Figures and Tables

**Table 1:** PPO hyperparameter settings.

Hyperparameter	Value
Batch Size	10
Buffer Size	100
Learning Rate	3.0 × 10^−4^
Epoch	3
Beta	5.0 × 10^−4^
Epsilon	0.2
Lambd	0.99
Max Steps	50,000

**Table 2:** Optimized hyperparameter settings.

Hyperparameter	Value
Batch Size	10
Buffer Size	100
Learning Rate	2.0 × 10^−4^
Epoch	3
Beta	5.0 × 10^−4^
Epsilon	0.2
Lambd	0.99
Max Steps	40,000

**Table 3:** Cumulative rewards comparation table.

Environment Setting	Cumulative Rewards Using Old Hyperparameter	Cumulative Rewards Using New Hyperparameter
Boxes with the same size	7.823	12.66
Boxes with different size	5.866	6.101
Smaller plane	56.03	238.1

**Table 4:** Total gaps of solutions result.

Solutions	1	2	3	4	5
Box 1	0.3659	0.3675	0.3678	0.3674	0.3672
Box 2	0.4071	0.4071	0.4071	0.4071	0.4071
Box 3	0.4076	0.4075	0.4075	0.4075	0.4075
Box 4	0.9169	0.9168	0.9168	0.9169	0.9169
Box 5	0.6786	0.6780	0.6774	0.6775	0.6776
Box 6	0.9997	1.0001	1.0004	1.0003	1.0003
Box 7	0.9635	0.9610	0.9601	0.9604	0.9607
Box 8	1.0913	1.0401	1.0131	1.0147	1.0163
Total Gaps	5.8307	**5.7780**	5.7502	5.7520	5.7537

**Table 5:** Study comparation.

	Heuristics	RL without Engine	This Study
Roll-out	Limited	Limited	Efficient
Stability	Prone to Issues	Prone to Issues	Enhanced
Arbitrary Shape Handling	Limited	Limited	Yes
Visualization	Limited	Limited	Intuitive
Ease of Modification	Difficult	Difficult	Easy

## Data Availability

Not applicable.
